# Human papillomavirus-related small cell carcinoma of the oropharynx: a case report and literature review

**DOI:** 10.1186/s41199-017-0022-4

**Published:** 2017-02-06

**Authors:** Marcelo Bonomi, Tamjeed Ahmed, David Warner, Joshua Waltonen, Christopher Sullivan, Mercedes Porosnicu, Katharine Batt, Jimmy Ruiz, James Cappellari

**Affiliations:** 1 Section on Hematology and Oncology, Wake Forest School of Medicine, Winston-Salem, NC 27157 USA; 2 Internal Medicine, Wake Forest School of Medicine, Winston-Salem, NC 27157 USA; 3 Department of Otolaryngology, Wake Forest School of Medicine, Winston-Salem, NC 27157 USA; 4 Department of Pathology, Wake Forest School of Medicine, Winston-Salem, NC 27157 USA

**Keywords:** Head and neck, Oropharynx, Chemotherapy, Radiation therapy

## Abstract

**Background:**

Small cell carcinoma (SCC) is a rare variant of head and neck cancer characterized by a high-grade neuroendocrine cancer with similar features to small cell lung carcinoma (SCLC). Human papillomavirus (HPV) is an increasingly recognized cause of head and neck cancer but usually associated squamous cell carcinoma of the oropharynx. In this report, we present the clinical presentation, diagnosis, and management of a patient with HPV-related SCC of the oropharynx that responded favorably to chemotherapy with cisplatin plus etoposide and concomitant radiation therapy, a regimen typically used in SCLC.

**Case presentation:**

We present a rare case of a 56-year-old man who presented with a three-month history of an enlarging left-sided neck mass. Imaging was consistent with a soft tissue density at the left tongue base, left level IIB nodal conglomerate, and multiple bilateral cervical lymph nodes, without evidence of distant metastasis. The patient underwent a core biopsy of the left neck level II node which read as a poorly differentiated neuroendocrine carcinoma consistent with small cell carcinoma. Polymerase chain reaction revealed that the tumor was positive for HPV16. The tumor was staged T1N2cM0 (stage IVA). He went on to receive four cycles of cisplatin and etoposide. On cycle two, he started radiotherapy to the oropharynx and involved neck nodes. He received a dose of 70 Gray (2 Gy/fraction) over a seven week-period. During the concomitant phase of chemo-radiation, the patient experienced grade IV mucositis, grade II nausea, and dehydration for which he received additional outpatient fluid and electrolyte replacement. Three months after completion of therapy, a PET/CT showed complete resolution of the tumor and metastatic lymph nodes along with no evidence of distant metastasis.

**Conclusion:**

Patients with HPV-related cancer of the oropharynx require identification of the small cell variant to optimize therapy and improve outcomes.

## Background

Small cell carcinoma (SCC) is a rare variant of head and neck cancer characterized by a high-grade neuroendocrine cancer with similar features to small cell lung carcinoma (SCLC). The most common location of head and neck SCC is the larynx, but it has also been reported in the sinonasal tract, salivary glands, trachea, oral cavity, and oropharynx [1]. Human papillomavirus (HPV) is an increasingly recognized cause of head and neck cancer with HPV-associated oropharyngeal squamous cell carcinoma (OPSqCC) being the most common variant. Although HPV positivity is a favorable prognostic factor in OPSqCC, HPV-related SCC of the oropharynx may share the same aggressive clinical features of SCLC [2]. In this report, we present a case of HPV-related SCC of the oropharynx that responded favorably to chemotherapy with cisplatin plus etoposide and concomitant radiation therapy, a regimen typically used in SCLC.

## Case presentation

A 56-year-old man with no history of tobacco use or alcohol consumption presented with a three-month history of an enlarging left-sided neck mass and worsening headaches. A positron emission tomography/computed tomography (PET/CT) showed an [18 F]fluorodeoxyglucose FDG-avid soft tissue density at the left tongue base measuring approximately 1.8 × 2 cm, a centrally hypodense hypermetabolic left level IIB nodal conglomerate measuring 3.6 × 4 cm, and multiple bilateral hypermetabolic cervical lymph nodes, without evidence of distant metastasis. Brain MRI was negative for brain metastasis.

The patient underwent a core biopsy of the left neck level II node which read as a poorly differentiated neuroendocrine carcinoma consistent with small cell carcinoma.

**Fig. 1:**
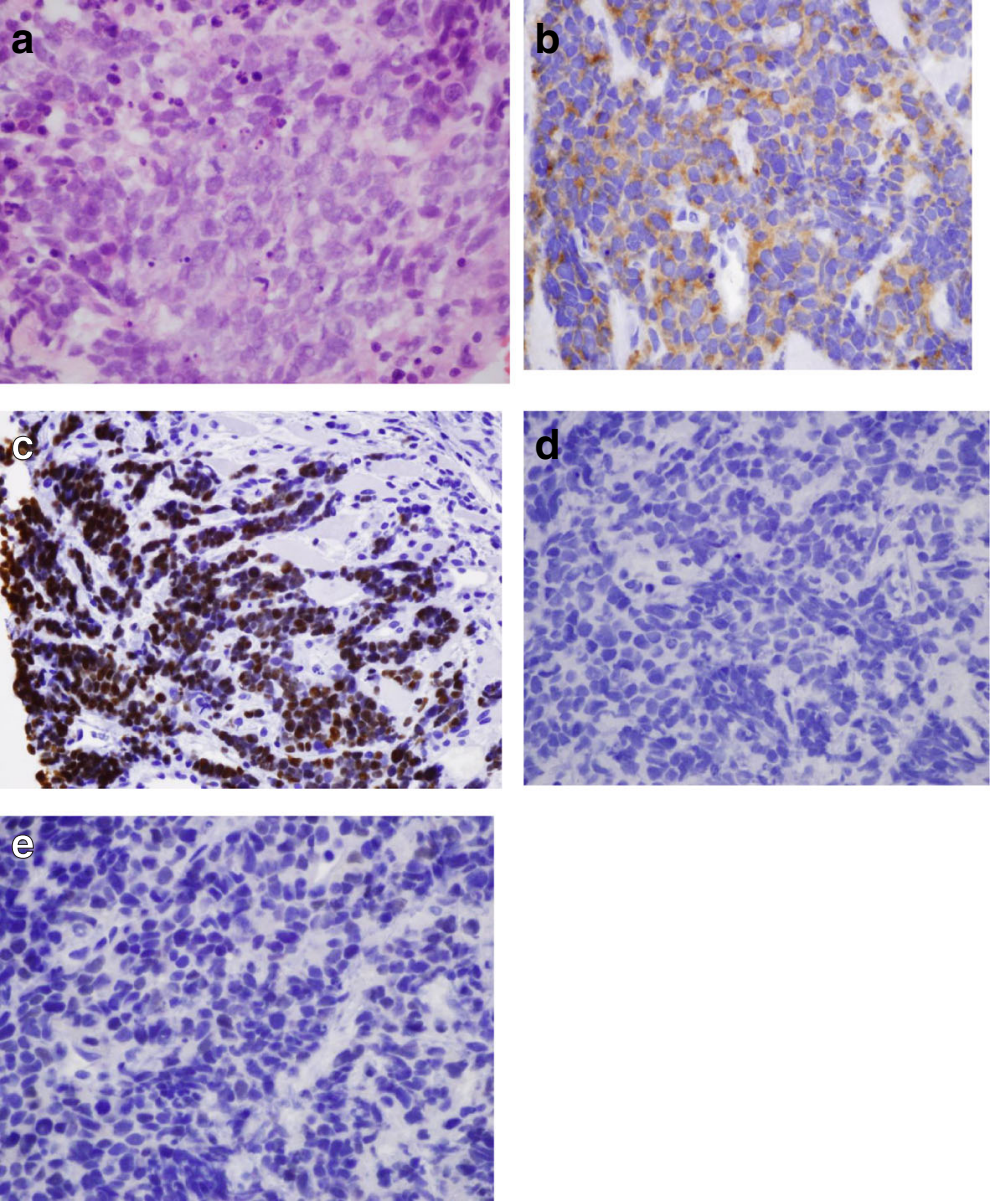
**a** Hematoxylin and Eosin tumor stain. **b** Synaptophysin tumor stain. **c** TTF-1 tumor stain. **d** Cytokeratin 5/6 stain. **e** P63 tumor stain

Core biopsy of the left neck level II node revealed sheets of malignant cells with small to intermediate-sized nuclei, indistinct nucleoli, and scant cytoplasm consistent with SCC. The tumor exhibited areas of necrosis as well as abundant mitotic figures and apoptotic bodies. The neoplastic cells were positive for cytokeratin AE1/AE3, synaptophysin, p16, and TTF-1 with a nuclear staining pattern; they were negative for cytokeratin 5/6, CAM 5.2, p63, chromogranin, CD56, and EBV (by in-situ hybridization) (Fig. 1).

The tumor was positive for p16, but the combined morphologic and immunophenotypic features argued against conventional HPV-associated OPSqCC. Polymerase chain reaction demonstrated that the tumor was positive for HPV16, negative for HPV18, 31, 33, 35, 39, 45, 51, 52, 56, 59, 66, and 68.

The tumor was staged T1N2cM0 (stage IVA). A percutaneous endoscopic gastrostomy tube (PEG) was placed before the beginning of treatment to meet his nutritional and hydration needs during treatment. He received four cycles of chemotherapy at 21 day-intervals. The chemotherapy regimen consisted of cisplatin 75 mg/m2 on day one and etoposide 80 mg/m2 on days one to three. On cycle two, day eight, he started radiotherapy to the oropharynx and involved neck nodes. He received a dose of 70 Gray (2 Gy/fraction) over a seven week-period. During the concomitant phase of chemo-radiation, the patient experienced grade IV mucositis, grade II nausea, and dehydration for which he received additional outpatient fluid and electrolyte replacement. Due to grade III neutropenia, the dose of cisplatin and etoposide was reduced by 25% during the last cycle of chemotherapy.

**Fig. 2:**
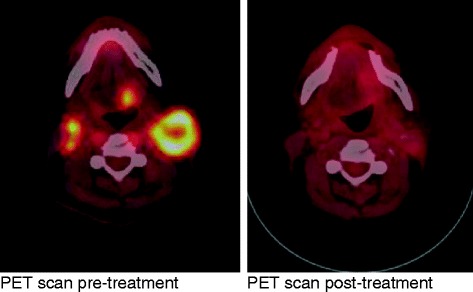
*Left* PET scan pre-treatment. *Right* PET scan post-treatment

Three months after completion of therapy, a PET/CT showed complete resolution of the tumor and metastatic lymph nodes along with and no evidence of distant metastasis (Fig. 2). He also had complete resolution of his mucositis and was able to resume a full oral diet resulting in removal of the PEG tube.

## Discussion and conclusions

Small cell lung cancer is distinguished by its rapid doubling time, high growth fraction, and early development of widespread metastases. Although highly responsive to chemotherapy and radiation initially, the majority of patients will eventually relapse with broadly resistant disease a few months to a year from initial therapy. SCLC occurs almost exclusively in smokers and appears to be most common in heavy smokers. Historically, SCLC has been rare in non-smokers, representing just 2.9% of lung cancer cases in women and none in men as reported in a case control series [3].

High risk HPV, particularly the 16 type, has been established as a causative agent for a significant proportion of OPSqCC. These tumors typically originate from the tonsillar crypts and have a characteristic appearance described as infiltration of the lymphoid stroma as lobules of immature basaloid cells with minimal cytoplasmic keratinization [4].

Routine HPV testing of OPSqCC has expanded the morphologic spectrum of HPV-related cases. These histologic variants include papillary, lymphoepithelial-like, basaloid squamous, and adenosquamous. Although these phenotypic variations may be diagnostically relevant, they do not appear to impact prognosis. The presence of HPV consistently imparts a favorable prognosis, even when detected in more aggressive phenotypes such as the basaloid squamous cell carcinoma [5].

For patients with HPV-related cancer of the oropharynx, recognition of the small cell variant and its distinction from HPV-related squamous cell carcinoma is important although not straightforward. Both tumor types share morphologic features that include small hyperchromatic cells with scant cytoplasm and comedonecrosis [6]. Absence of p63 is often used to differentiate SCLC from squamous cell carcinoma of the lung [7, 8] yet in one case series 4 of 8 tested oropharyngeal SCCs were p63 positive suggesting poor reliability of this biomarker. Cytokeratin 5/6 on the other hand, seems to be a more reliable distinguishing marker [2]. In contrast to OPSqCCs that are consistently cytokeratin 5/6 positive, all SCCs are cytokeratin 5/6 negative. A minority of oropharyngeal SCC is TTF-1 positive which seems to signify high specificity for SCC in this setting [9]. In general, all SCCs demonstrate immunohistochemical evidence of neuroendocrine differentiation namely synaptophysin and chromogranin positivity [2]. In the case of SCCs of the oropharynx, p16 positivity may not be a reliable surrogate marker for HPV infection given that in one study, 2 of 4 SCCs were p16 positive but HPV negative by in-situ hybridization. This is likely consistent with the finding of p16 positivity in many sites of SCC due to mechanisms unrelated to HPV infection [2, 10].

In SCLC, the most frequently used chemotherapy regimen is a platinum (either cisplatin or carboplatin) with etoposide based upon the clinical activity and toxicity profile of the platinum agent. Because both agents possess little mucosal toxicity, limited risk for interstitial pneumonitis, and modest hematologic toxicity, platinum plus etoposide is the regimen of choice to use with concurrent chest radiation therapy in patients with limited stage SCLC. Due to its proven activity in SCLC and its favorable toxicity profile when used in combination with external radiation therapy, we decided to use this regimen for four cycles with concomitant definitive radiation. The patient’s HPV status did not influence our treatment decision. We hope that HPV positivity portends a favorable prognosis in this case but from reviewing the few cases in the literature, it appears that these patients clinically behave similar to the aggressive nature of SCLC [11]. Further studies are needed to determine the pathophysiology of how HPV results in SCC, only then can potential molecular targets be discovered which will lead to the development of targeted treatments. In this patient, the regimen was proven to be active with manageable toxicities, but longer follow-up will be needed to determine true efficacy.

In conclusion, patients with HPV-associated cancer of the oropharynx require an accurate diagnosis to distinguish the more common squamous cell carcinoma from the rare small cell variant. This identification is vital in order to optimize therapy and improve treatment outcomes.

## Abbreviations

HPV: Human papillomavirus
OPSqCC: Oropharyngeal squamous cell carcinoma
PET/CT: Positron emission tomography/computed tomography
SCC: Small cell carcinoma
SCLC: Small cell lung cancer
